# Spatial Distribution of (R)-salbutamol in Rat Brain Following Nasal and Intravenous Administration Using DESI-MS

**DOI:** 10.3390/pharmaceutics12010035

**Published:** 2020-01-02

**Authors:** Rui Zhang, Jie Wu, Siyu Liu, LiangJun Deng, Junhua Hu, Xi Chen, Wen Tan

**Affiliations:** 1 Institute of Biomedical and Pharmaceutical Sciences, Guangdong University of Technology, Guangzhou 510006, Guangdong, China; scut_gdut@163.com (R.Z.); lsy13113955174@163.com (S.L.); dengliangjun@mail2.gdut.edu.cn (L.D.); hu_jun_hua@163.com (J.H.); ann_chen0806@163.com (X.C.); 2 School of Pharmacy, Jinan University, Guangzhou 510632, Guangdong, China; wjhgg2005@163.com; 3 YZ Health-tech Inc., Hengqin District, Zhuhai 519000, Guangdong, China; 4 Jeffrey Cheah School of Medicine and Health Sciences, Monash University Malaysia, Bandar Sunway 47500, Selangor Darul Ehsan, Malaysia

**Keywords:** Parkinson’s disease, β2-adrenergic receptor, salbutamol, nasal administration, desorption electrospray ionization

## Abstract

Recent studies have shown that β2-Adrenoreceptor is a regulator of the a-synuclein gene driving risk of Parkinson’s disease. The β2-AR agonist (R)-salbutamol, eutomer of rac-salbutamol, may hold therapeutic potential for Parkinson’s disease (PD) following nasal administration. In this study, we use desorption electrospray ionization mass spectrometry (DESI-MS) to analyze spatial distribution of (R)-salbutamol in rat brain following nasal and intravenous administration. Here, we report that (R)-salbutamol efficiently deliver to the brain and had more drug dosage exposure in rat’s brain through nasal route administration than that of intravenous route administration. In conclusion, administering (R)-salbutamol through nasal route of administration may hold advantages in improving spatial distribution and increased exposure of drug in brain.

## 1. Introduction

**Figure 1:**
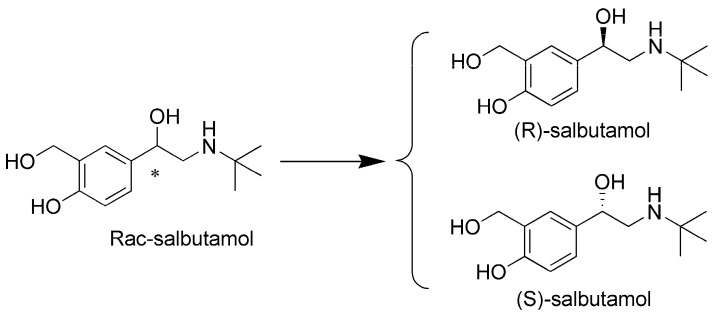
Structure of rac-salbutamol, (R)-salbutamol and (S)-salbutamol.

Salbutamol is a short-acting β2-AR agonist and it is used for respiratory diseases (structure shown in Figure 1) [1]. Recent studies have demonstrated that use of salbutamol is associated with a decreased Parkinson’s disease (PD) risk (β2-Adrenoreceptor is a regulator of the a-synuclein gene driving risk of Parkinson’s disease) [2,3]. Salbutamol is a racemic mixture of (S)-salbutamol and (R)-salbutamol and (R)-salbutamol is the eutomer (structure shown in Figure 1) [4]. Binding of (R)-salbutamol and S-salbutamol with β2-receptor has opposite effects on calcium concentration, bronchoconstriction, airway responsiveness and eosinophils [5,6,7,8]. Moreover, (R)-salbutamol can activate adenylate cyclase and increase the content of cAMP in cells, and increased cAMP will inhibit the release of inflammatory factors produced by mast cells and eosinophils [9]. (S)-salbutamol, meanwhile, will inhibit adenylate cyclase, cause the decrease of cAMP content, and have the opposite effect with (R)-salbutamol [10]. Thus, β2-AR agonist used (R)-salbutamol may be a little more appropriate for PD treatment compared with (S)-salbutamol. However, salbutamol has not been specifically developed for PD [11]. Results from rat experiment showed that salbutamol has poor property to penetrating the blood brain barrier (BBB) and reached brain concentrations amounting to about 5% of the plasma concentrations after 10 mg/kg i.v. [12].

Intranasal administration is a route of drug administration of delivering active pharmaceutical ingredients to the nasal cavity, systemic and brain [13]. The anatomical structure of the nasal cavity is closely related to the brain [14]. Nasal cavity consists of nasal vestibule, respiratory region and olfactory region. After intranasal administration, part of the drug passes through the olfactory mucosa, along the tissues surrounding the olfactory nerve tract and the axons of olfactory neurons to the cerebrospinal fluid or brain [15], the other part enters the systemic circulation through the capillary-rich respiratory region [16]. Nasal administration has the advantages of high bioavailability, fast acting, simple administration and brain targeting ability. Many effects have been done to explore the nasal route for delivery of drugs to the brain via a specific site, the olfactory region [13]. A recent study showed that intranasal administration can significantly improve the bioavailability of buspirone, and brain targeting rate was 75.77% [17]. Moreover, intranasal administration of carbamazepine is an effective way to treat epilepsy, it can be used not only for long-term administration, but also for acute conditions [18]. We predicted that more salbutamol might reach to brain through the nasal route than intravenous administration. At present, there has been a comparison of the efficacy and bioavailability of salbutamol after intranasal administration and intravenous administration. Anwar A. Hussain et al. founded that albuterol is effective in providing bronchoprotection when delivered intranasally to guinea pigs. Intranasal albuterol has longer effect compared with intravenous [19]. Whereas salbutamol is mainly used in the treatment of respiratory diseases such as asthma. There are not many reports about its brain targeted ability by nasal administration.

Desorption electrospray ionization mass spectrometry (DESI-MS) is an efficient method to detect small-molecule on biological tissues, and provides information on the spatial distribution of molecules at the surfaces [20]. DESI-MS could be used to explore spatial distribution of salbutamol in brain following nasal and intravenous administration. On this basis, we confirmed spatial distribution of (R)-salbutamol in rat brain through nasal and intravenous administration using DESI-MS.

## 2. Materials and Methods

### 2.1. Chemicals

Ultra pure water was from Millipore (Danvers, MA, USA), ULC/MS grade methanol was purchased from Thermo Fisher Scientific (Waltham, MA, USA), 99% formic acid was purchased from J&K Scientific LTD (Beijing, China), microscopic glass slides was purchased from Citotest Labware Manufacturing Co., Ltd. (Nanjing, China), levosalbutamol sulfate was provided by Key-pharma Biomedical Inc. (Dongguan, China), leucine enkephalin (LE) was obtained from Waters Corporation (Milford, MA, USA), chloral hydrate was purchased from Aladdin Industrial Corporation (Shanghai, China), sodium chloride inject was purchased from Guangxi Yuyuan Pharmaceutical Co., Ltd. (Guangxi, China). All chemicals and reagents were used without further purification.

### 2.2. Animals

Male rat (Sprague-Dawley, SD), which their weight was about 180–200 g were purchased from the Laboratory Animal Center of Southern Medical University (Guangzhou, China). Cultural conditions were controlled at temperature: 25 ± 2 °C, humidity: 60 ± 5%, 12 h dark-light cycle. Rearing the animals in acrylic cages. Food and water are adequately provided. We changed the bedding material once every three days. All experimental studies were approved by the Animal Ethics Committee of South China University of Technology (Approval ID: 20181526091; Date: 21 May 2018).

### 2.3. Tissue Collection and Preparation

**Figure 2:**
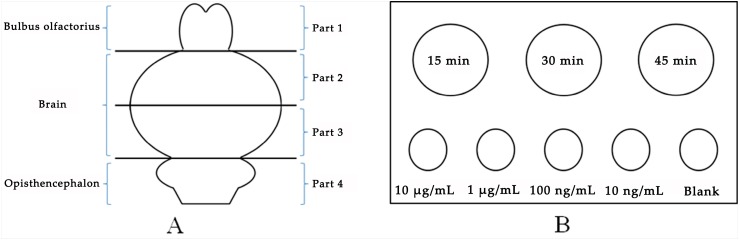
Sample description of tissue sections. (**A**) Description of the four parts of the brain and (**B**) description of the brain slices of three time points and standards of different concentrations.

SD rats were divided into two groups: intravenous and nasal administration (nine SD rats per group). Each group was divided into three administration time points group: 15 min, 30 min and 45 min (three SD rats per time points group). The dosage of intravenous and nasal administration groups was the same (1.6 mg, in this paper, our aim was to confirm the difference of spatial distribution in rat brain for (R)-salbutamol between nasal and intravenous administration. It is easy to find the difference between these two routes using 1.6 mg/animal dosing. Besides, this dosing was safe for animals as previous study [12] using 10 mg/kg i.v. to study kinetics and distribution of salbutamol in the rat brain and our dosing was lower than 10 mg/kg). Different from intravenous administration, the volume of solution for nasal administration should not be too large. We prepared two concentrations of (R)-salbutamol solution with normal saline, one was 40 mg/mL for nasal administration and another was 10 mg/mL for intravenous administration. We used pipette to nasal administration (5 µL per nostril at a time, four times in total). We used 1 mL syringe to tail vein administration (0.16 mL per SD rat). After administration heart perfusion with saline was implemented. Then the brain and olfactory bulb were taken. When the tissue was firmly frozen, the tissues divided into four parts (Figure 2A) cryo-sectioned using a freezing microtome into 20 µm thick tissue sections. (R)-salbutamol of different concentration (10 μg/mL, 1 μg/mL, 100 ng/mL, 10 ng/mL and blank) was mixed with brain homogenate as a relative quantitative standard. The placement of standard and brain slices on microscopic glass slides was shown in Figure 2B. High resolution mass spectrometry (HRMS) with a DESI source was used to scan slices and relative quantitative standards, and the results were analyzed to compare the differences of drug concentration and distribution.

### 2.4. DESI Data Acquisition and Processing

The angle between the sprayer and the sample surface was set to 55°. The electrospray solvent consisted of methanol/water/formic acid (95:5:0.1 *v*/*v*), and the flow rate was used at 2 μL/min. Nitrogen was used as the nebulizing gas at a pressure of 0.5 MPa. DESI-MSI was performed using a DESI q-TOF MS, which made by Waters Corporation (SYNAPT G2si HDMS). The spray capillary voltage was set to 4.5 kV (positive ion mode), the Cone voltage was set to 60 V, and the ion source temperature was set to 100 °C. Mass spectra were acquired using positive ionization mode and the range of m/z was from 50 to 1000. Number of most intense peaks it collected was the top 1000. All m/z values were extracted with a mass window of 0.002 Da. The Lock Mass was 556.2772 m/z. These conditions were constant for all the experiments conducted below.

#### 2.4.1. Step to Get the Result of Imaging

Prior to tissue imaging, we intended to find the molecular ion of (R)-salbutamol. The (R)-salbutamol signal was extracted from high concentration standard (10 μg/mL) and corrected with LE. HDImaging software provides extended functionality to support the acquisition and processing of data from DESI imaging experiments. It enables images showing spatial distribution of components across a sample surface to be displayed, and the components to be identified and analyzed. We used it to process and image MS data.

#### 2.4.2. Spatial Distribution Mapping of (R)-Salbutamol in the Rat Brain

All the raw data were analyzed based on method 2.4.1 and spatial distribution mappings of (R)-salbutamol were shown in the result of 3.2. Then we could compare spatial distribution of (R)-salbutamol in the rat brain following nasal and intravenous administration.

#### 2.4.3. Spatial distribution mapping of cleavage molecule of (R)-salbutamol in the Rat Brain

In order to further confirm that the molecule we found was salbutamol, we searched for its cleavage molecule.

## 3. Results and Discussion

### 3.1. Step to Get the Result of Imaging

**Figure 3:**
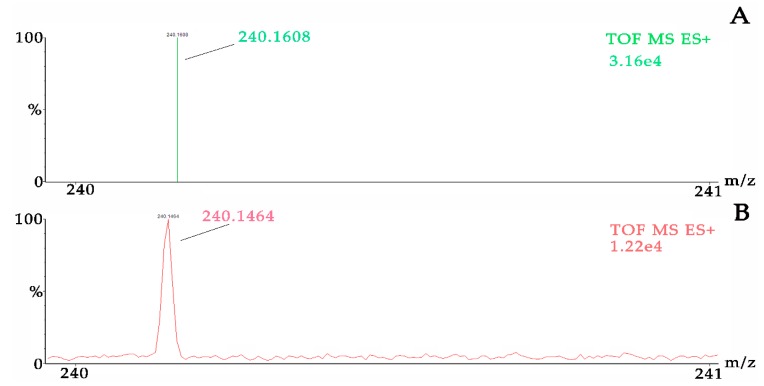
Original and corrected m/z values of [(R)-salbutamol + H]^+^ in part 1 of nasal administration. (**A**) Description of the mass after corrected and (**B**) description of the mass before corrected.

In positive ion mode, previous study showed that high signals of the protonated molecular ion of salbutamol (m/z 240.1) could be detected by ESI source [21]. The exact mass of [M + H]^+^ was 240.1594. Taking part 1 of nasal administration for example, we extracted m/z values of 240.159 from the total ion current (TIC) result in the location of 10 μg/mL standard part and then used 556.2771 (LE) to adjust the results. Ion at m/z 240.1608 from the range of 240 to 241 was selected and its intensity is 3.16e4 (Shown in Figure 3).

**Figure 4:**
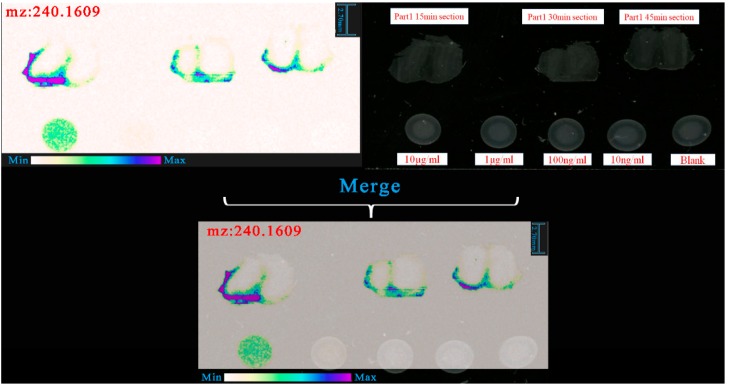
Method to confirm the protonated molecular ion of (R)-salbutamol.

Then HDI software was used to analyze the raw data and the range of processing was from 200 to 300 Da. Based on HDI software generated results, we found that the intensity of 240.1609 in 10 μg/mL plaque was higher than that of other plaques at the area of standard samples. Combined with exact mass of [M + H]^+^, 240.1609 was the protonated molecular ion of salbutamol. This result was merged with original section and we observed the distribution of (R)-salbutamol in the rat’s brain (shown in Figure 4).

### 3.2. Spatial Distribution Mapping of (R)-Salbutamol in the Rat Brain

**Figure 5:**
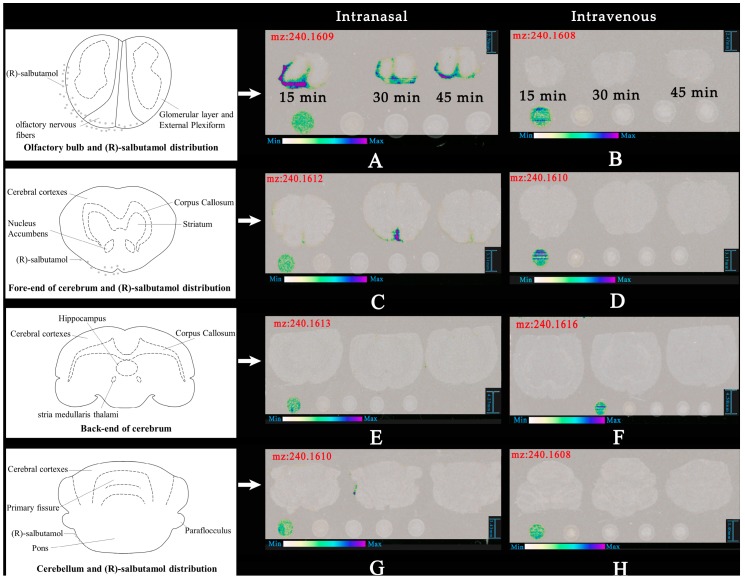
Spatial distribution of (R)-salbutamol in the section of rat brain. (**A**) and (**B**) are the olfactory bulb area of intranasal administration and intravenous injection of (R)-salbutamol respectively. Similarly, (**C**) and (**D**) are the fore-end of the cerebrum area. (**E**) and (**F**) are the back-end of the cerebrum area. (**G**) and (**H**) are the cerebellum area.

Compared with the part 1 of nasal and intravenous administration (shown in Figure 5A,B), nasal administration facilitated the delivery of (R)-salbutamol to the brain significantly than through intravenous administration. In the nasal administration group, the signal response intensity in area 1 (olfactory bulb) of DESI scanning results of rat brain slices was the highest. Due to the existence of blood–brain barrier, at least 98% of the alternative drugs for central nervous system therapy cannot reach the brain [22]. Immediately after nasal administration, drugs enter the brain through three major pathways. 1) Olfactory nerve pathway and 2) epithelial pathway of olfactory mucosa. 3) Blood circulation pathways [23]. In nasal administration group, DESI scan results in the first area were consistent with the study [15].

**Figure 6:**
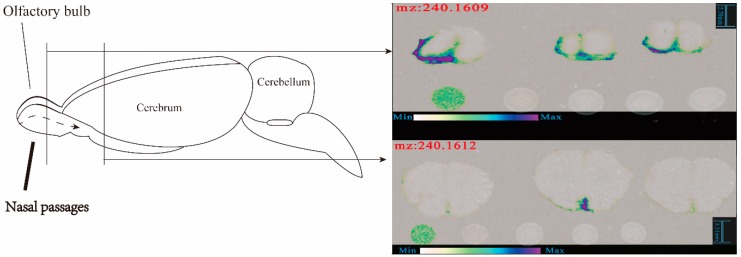
The pathway of (R)-salbutamol into brain after nasal administration.

In part 2 (fore end of cerebrum), (R)-salbutamol was detected in the section of nasal administration group but not in the sections of intravenous administration group under the same conditions (shown in Figure 5C,D). For part 3 (back end of cerebrum) There was no significant difference in detecting (R)-salbutamol between nasal and intravenous administration (shown in Figure 5E,F). As shown in Figure 5G,H, only in the section of nasal administration group at 30 min detected low level of (R)-salbutamol but not in intravenous administration group. Figure 5 shows that in nasal administration group, the response intensity of brain slice signal at 30 min time point is stronger in area 2 and 4. However, at 15 min time point area 1 had the strongest intensity. The first, second and fourth brain slices of nasal administration showed corresponding signals, however, no corresponding signals were observed in four brain slices of intravenous administration under the same scanning conditions. As shown in Figure 6, the act of rapidly delivering (R)-salbutamol from the nasal cavity to the brain after nasal administration could be mainly due to its intracellular and extracellular pathways surrounding peripheral olfactory nervous system. Whereas we found that the signal for (R)-Salbutamol was seemingly trapped into the arachnoidal cerebrospinal fluid (CSF) region, and it seemed decreasing in intensity from 15 to 45 min. There is no sign that there was not any BBB penetration in the DESI result of this experiment. For this result, more experiments are needed to confirm whether (R)-salbutamol is able to cross the BBB. At first, we used DESI to explore spatial distribution of (R)-salbutamol in the rat brain. A previous study has shown that (R)-salbutamol was mostly metabolized via isomerization, oxidation, reduction, glucuronidation and sulfation pathways in vivo [24]. In addition, there is no previously evidence indicate that (R)-salbutamol can be delivered through passive and active transport to cross tissue membrane. It is first time we demonstrate that (R)-salbutamol can be delivered into brain via nasal related pathway.

Microdialysis or direct CSF sampling can also provide the information that a comparison of the amount of (R)-salbutamol delivered to brain. However, we think the DESI method could also provide a spatial distribution of (R)-salbutamol.

In this experiment, we have set standard samples on the section. It is difficult to confirm the absolute concentration in the brain tissue using our methods. However, the standard samples could not be omitted. There are two functions of the standard samples. The first function was to find the target molecule weight. By setting the standard samples, it is convenient to confirm the ion what we need and modulate the parameters of analyzing software. The second function is to compare the relative amount between different sections.

### 3.3. Spatial Distribution Mapping of Cleavage Molecule of (R)-Salbutamol in the Rat Brain

**Figure 7:**
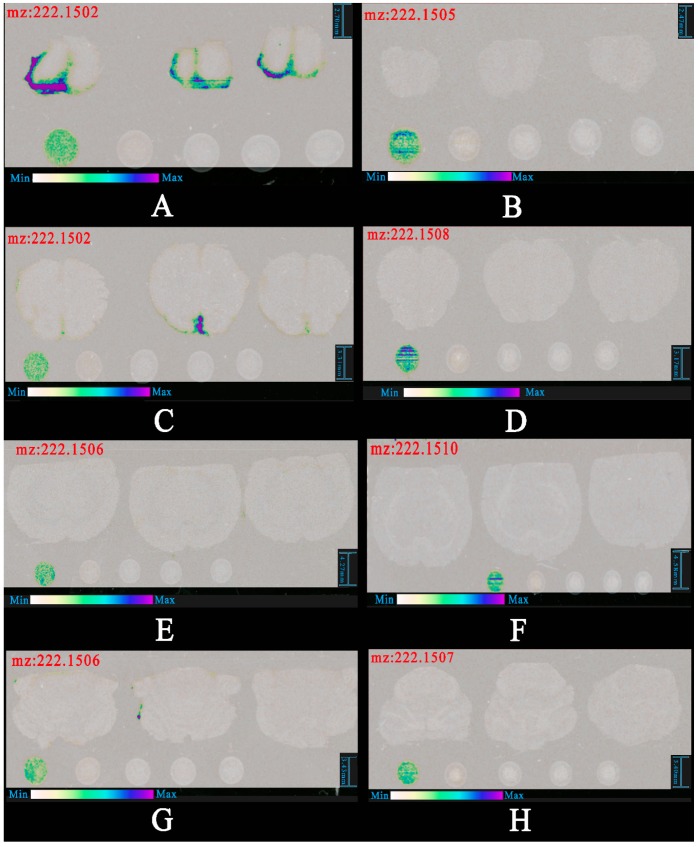
Spatial distribution of m/z at 222.15 in the section of rat brain. (**A**) and (**B**) are the olfactory bulb area of intranasal administration and intravenous injection of (R)-salbutamol respectively. Similarly, (**C**) and (**D**) are the fore-end of the cerebrum area. (**E**) and (**F**) are the back-end of the cerebrum area. (**G**) and (**H**) are the cerebellum area.

**Figure 8:**
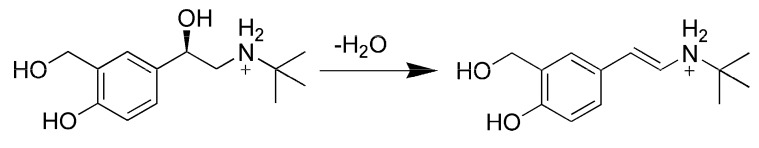
Structures of [(R)-salbutamol + H]^+^ and [(R)-salbutamol-H2O + H]^+^.

Through analysis the imaging results of all ion, we found that spatial distribution mapping of m/z at 222.15 was the same with the protonated molecular ion of (R)-salbutamol (shown in Figure 7). We predicted that this molecule might be the protonated molecular ion of (R)-salbutamol losing H_2_O (shown in Figure 8).

**Figure 9:**
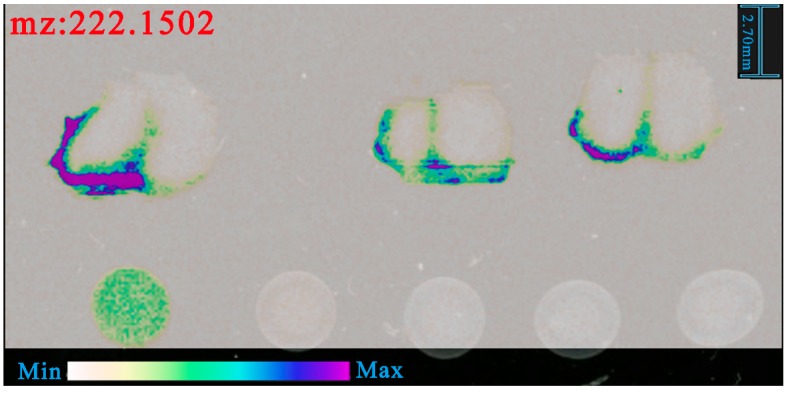
Spatial distribution of m/z at 222.1502 in part 1 of nasal administration.

The exact mass of [(R)-salbutamol-H_2_O + H]^+^ was 222.1489. Taking part 1 of nasal administration as an example, we found that the distribution of m/z at 222.1502 was similar to m/z at 240.1609 (shown in Figure 9). Difference value between m/z at 222.1502 and m/z at 240.1609 was 18.0107, which was the exact mass of H_2_O (calculated m/z of H_2_O was 18.0106). So, this result was confirmed to be [(R)-salbutamol-H_2_O + H]^+^.

**Figure 10:**
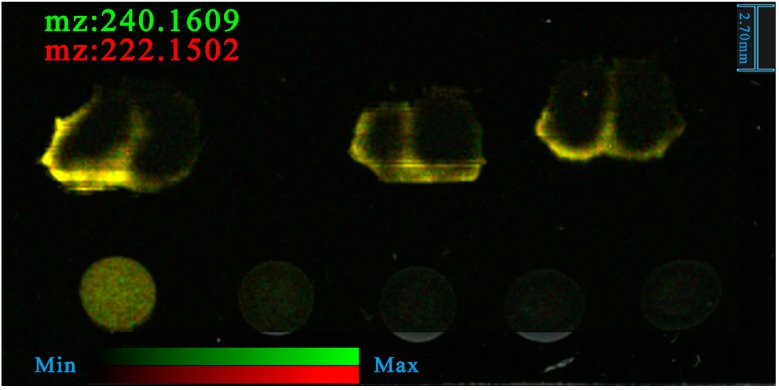
Spatial distribution of m/z at 240.1609 and m/z at 222.1502 in part 1 of nasal administration.

We merged the imaging results of m/z at 240.1609 and m/z at 222.1502 and found that the spatial distribution of these two ions were similar (shown in Figure 10). Thus, imaging result of 222.15 could be used to verify the distribution of (R)-salbutamol. From the results of 222.15 and 240.16, we confirmed that (R)-salbutamol was easier delivered to the brain and reached more dosage through nasal route than that of intravenous administration.

## 4. Conclusions

In this study, we reported the spatial distribution mapping of (R)-salbutamol in the rat brain following nasal and intravenous route of administration using DESI-MS. (R)-salbutamol efficiently was delivered to the brain and significance more drug exposure through nasal administration compared with intravenous administration. In addition, this experiment shows that DESI-MS is a useful tool for comparing and analyzing the delivery effect of different administration routes.
